# Designed, Programmable Protein Cages Utilizing Diverse Metal Coordination Geometries Show Reversible, pH‐Dependent Assembly

**DOI:** 10.1002/marc.202400712

**Published:** 2024-12-16

**Authors:** Norbert Osiński, Karolina Majsterkiewicz, Zuzanna Pakosz‐Stępień, Yusuke Azuma, Artur P. Biela, Szymon Gaweł, Jonathan G. Heddle

**Affiliations:** ^1^ Malopolska Centre of Biotechnology Jagiellonian University Gronostajowa 7A Kraków 30387 Poland; ^2^ Doctoral School of Exact and Natural Sciences Jagiellonian University Łojasiewicza 11 Kraków 30384 Poland; ^3^ Postgraduate School of Molecular Medicine ul. Żwirki i Wigury 61 Warsaw 02091 Poland; ^4^ School of Biological and Biomedical Sciences Durham University Durham DH1‐3LE UK; ^5^ National Synchrotron Radiation Centre SOLARIS Czerwone Maki 98 Kraków 30392 Poland

## Abstract

The rational design and production of a novel series of engineered protein cages are presented, which have emerged as versatile and adaptable platforms with significant applications in biomedicine. These protein cages are assembled from multiple protein subunits, and precise control over their interactions is crucial for regulating assembly and disassembly, such as the on‐demand release of encapsulated therapeutic agents. This approach employs a homo‐undecameric, ring‐shaped protein scaffold with strategically positioned metal binding sites. These engineered proteins can self‐assemble into highly stable cages in the presence of cobalt or zinc ions. Furthermore, the cages can be disassembled on demand by employing external triggers such as chelating agents and changes in pH. Interestingly, for certain triggers, the disassembly process is reversible, allowing the cages to reassemble upon reversal or outcompeting of triggering conditions/agents. This work offers a promising platform for the development of advanced drug delivery systems and other biomedical applications.

## Graphical abstract

A hyperstable artificial protein cage can be disassembled on demand under specific conditions such as pH below 7. The cage reassembles when conditions are reversed.

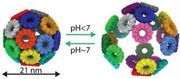

## 1 Introduction

Protein cages are hollow, nanometric structures made from numerous copies of protein subunits that self‐assemble into defined sizes and shapes. They are of interest as platforms for drug delivery or vaccines, owing to their potential for carrying cargo in their lumens and displaying multiple ligands on their external surfaces^[^ ^1^, ^2^, ^3^, ^4^, ^5^, ^6^ ^]^ A challenge for delivery applications lies in their highly stable assembly. Consequently, disassembly typically requires harsh conditions, for instance, pH ≤2 in the case of ferritin, a naturally occurring protein cage commonly engineered for multiple biotechnological purposes.^[^ ^7^ ^]^ This hampers packaging and release of cargo molecules while retaining their functionality, particularly for biological guests such as proteins and RNAs, for which proper folding is usually sensitive to the environment. In protein cage assemblies, constituent proteins are held in place by multiple interactions at protein‐protein interfaces, typically a mixture of electrostatic and hydrophobic interactions. These interfaces can be complex, consequently making it difficult to redesign them to control assembly characteristics.

**Figure 1:**
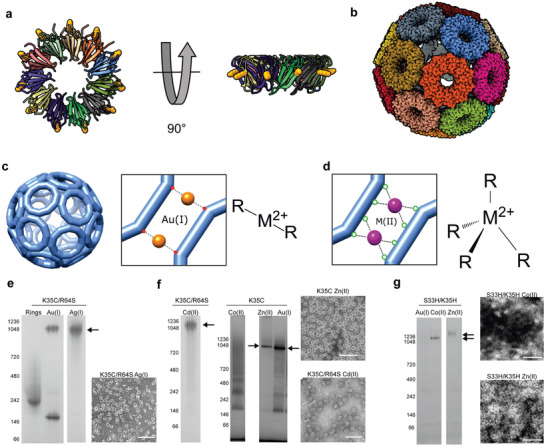
TRAP‐cage design and formation with various metals. a) Structure of a TRAP ring (pdb 1qaw) shown in two orthogonal views and in cartoon format with each monomer a different color. Position 35 is shown as yellow spheres. b) Structure of the 24‐ring TRAP‐cage (pdb 6rvv) assembled in the presence of Au(I) shown in space‐filling representation with each ring a different color. c) Schematic showing ring‐ring interface in TRAP^Au^‐cages. Left‐hand side represents the overall structure of the cages, with close‐up view (middle) of the ring‐ring interface showing linear coordination of gold between opposing thiols of residue 35 (represented by red dots). Right shows an idealized representation of the linear coordination. d) Close‐up of the ring‐ring interaction design for tetrahedral metal coordination with (right) idealized arrangement of a single tetrahedral sphere around a metal ion also shown. In our designs R groups may consist of 4 × His or 2 × His and 2 × Cys. e) Native PAGE of TRAP‐cage formation via linear coordination using TRAP(K35C/R64S) in the presence of metal as indicated. Arrows indicate position of bands corresponding to TRAP‐cages. Adjacent negative stain TEM shows TRAP^K35C^ after incubation with indicated metal. f) Native PAGE of TRAP‐cage formation with metals expected to have higher coordination numbers, using TRAP^K35C^ or TRAP ^K35C/R64S^ in the presence of indicated metals. Adjacent negative stain TEM shows TRAP^K35C^ after incubation with indicated metals. g) Native PAGE of TRAP‐cage formation with metals expected to have higher coordination numbers using TRAP^S33H/K35H^ in the presence of metal as indicated. Adjacent negative stain TEM shows TRAP^S33H/K35H^ after incubation with indicated metals. Numbers on the left of each gel indicate position of molecular weight marker bands (kDa). Arrows indicate position of bands corresponding to TRAP‐cages. Scale bars = 100 nm.

A prominent strategy for controlling protein cage assembly dynamics is to connect building blocks through metal ion bridging so that disassembly can be induced by the addition of competing ligands. This has been shown with modified ferritin variants possessing a protein‐protein interface reengineered to assemble via Cu(II) coordination and at a reengineered protein‐protein interface, where triggered disassembly was achieved by the addition of ethylenediamine tetraacetate (EDTA).^[^ ^8^ ^]^ A similar approach has also been employed with proteins that do not form hollow structures in naturally occurring states, but assemble into cage‐like architectures upon engineering/modification, referred to as “artificial protein cages”. Such examples include cytochrome cb_562_ which was modified with ligand moieties to form hexameric and dodecameric cages upon addition of Fe(II) and Zn(II).^[^ ^9^ ^]^ We have shown that cysteine‐modified variants of the trp RNA binding attenuation protein,^[^ ^10^, ^11^, ^12^ ^]^ assemble into cage‐like structures with Au(I) or Hg(II), called TRAP‐cage,^[^ ^11^, ^12^ ^]^ disassembly of which can be induced by thiol‐ or phosphine‐containing compounds (**Figure** **1** a,b).

TRAP^[^ ^10^, ^13^, ^14^, ^15^, ^16^, ^17^, ^18^ ^]^ has proved a useful building block for construction of artificial nanostructures.^[^ ^19^, ^20^, ^21^, ^22^ ^]^ The wild‐type protein forms a ring‐shaped assembly comprising 11 identical monomers. Its cage‐forming ability was first demonstrated via addition of Au(I) to TRAP variants containing an introduced cysteine at the rim region, TRAP^K35C^.^[^ ^11^, ^23^ ^]^ Changing residue arginine 64 to serine was previously introduced for modulating interaction of TRAP rings with gold nanoparticles^[^ ^22^ ^]^ but was found to have no significant effect on ability of TRAP rings to form cages. Variants with and without the R64S change are used interchangeably. The resulting cage‐like structures are composed of 12 or 24 copies of the rings connected to each other via S‐Au(I)‐S coordination (Figure 1c), referred to as TRAP^Au(I)^_12_‐ or TRAP^Au(I)^_24_‐cage, respectively, where the number of rings depends on the relative stoichiometry of protein to gold ions. TRAP^K35C^‐cage formation can occur with bismaleimide cross‐linkers, instead of Au(I), whereby disassembly can be programmed according to the properties of the linker used.^[^ ^24^ ^]^ TRAP‐cages are readily modified using genetic and chemical methods, allowing guest packaging in the lumen and modification of the exterior.^[^ ^25^ ^]^ Together with inducible disassembly, this makes them potent vehicles for intracellular delivery of cargo molecules.^[^ ^26^ ^]^

Challenges to some biomedical applications of TRAP‐cages can arise from metal ion bridges that, depending on the metals used, may be expensive and not biocompatible. Molecular cross‐linkers have shown promise as alternatives in this regard, though to date they have shown somewhat reduced cage‐formation efficiency compared to that seen with Au(I). Therefore, it would be beneficial to explore different metal ions for TRAP‐cage formation. Here, we report variants of TRAP which can assemble into TRAP‐cages mediated by Co(II) or Zn(II). All the resulting cages show a similar size and shape but different stability against external stimuli such as chelating agents or pH change, providing a library of off‐the‐shelf cages with varying disassembly properties, selectable depending on required applications.

## 2 Results

### 2.1 TRAP‐Cage Assembly with Ag(I)

The original TRAP^Au(I)^‐cage was formed by Au(I) (or Hg(II)) bridging between thiol groups of adjacent ring‐shaped proteins via linear coordination.^[^ ^12^ ^]^ Therefore, we first investigated if cage formation occurs with another metal ion, Ag(I), which adopts a similar linear coordination. TRAP^K35C/R64S^ was mixed with silver nitrate in aqueous solution and analyzed by native PAGE and negative‐stain transmission electron microscopy (TEM), showing the formation of hollow structures with the size and shape similar to TRAP^Au(I)^_24_‐cage (Figure 1e).

### 2.2 TRAP‐Cage Assembly via Non‐Linear Coordinating Metals

In order to address the effect of metals expected to have higher coordination numbers we carried out initial experiments using cadmium, a neighbor of silver on the periodic table. This showed that TRAP‐cages were able to form upon the addition of cadmium nitrate, providing Cd(II) (Figure 1f), which is typically found with a coordination number of 4–6.^[^ ^27^ ^]^ These results suggested that linear coordination is not essential, but rather that a strong bonding between ligands and metal ions is sufficient for the formation of cage‐like structures.

Despite successful cage formation, Ag(I) and Cd(II) are likely unsuitable for biomedical applications due to their toxicity. Therefore, again using TRAP^K35C^, we next performed analogous experiments with metal ions that exist in biological systems, Zn(II) and Co(II).^[^ ^28^ ^]^ Efficient formation of cage‐like structures with a morphology similar to TRAP^Au(I)^_24_‐cage was observed with Zn(II) (Figure 1f), whereas this is not the case with Co(II) (Figure 1f). These results further highlight the importance of high affinity to thiol ligands in successful cage‐formation of the cysteine‐modified TRAP.

In order to target metal ions with a preference for higher coordination, we reengineered the metal‐binding site of TRAP. Based on the crystal structure of wild‐type TRAP, a histidine residue was introduced at position 33 of the TRAP^K35C^ variant, yielding TRAP^S33H/K35C^, where the histidine and cysteine residues are located at *i* and *i+2* positions of the *β*‐sheet motif around the rim of the ring‐shaped structure. The cysteine residue at position 35 of TRAP^S33H/K35C^ was further changed to histidine, resulting in another variant, TRAP^S33H/K35H^, possessing a pair of imidazole ligands. Individual monomer units of these variants were expected to provide two ligands for metal ion coordination (Figure 1c,d).

We tested these new variants for cage formation in the presence of Co(II) and Zn(II) compared to Au(I). When the TRAP^S33H/K35H^ protein was mixed with Co(II) or Zn(II), it showed the formation of pseudospherical cages with a diameter of ≈22 nm, similar to TRAP ^Au(I)^_24_‐cage (Figure 1g). Au(I)‐mediated cage formation was not observed with this variant, likely due to the lower affinity of gold ions to imidazole than thiol. Meanwhile, TRAP^S33H/K35C^ showed cage formation as for TRAP^K35C^, i.e., cage formation occurred with Zn(II) and Au(I), but not with Co(II) (Figure S1, Supporting Information). The cage‐formation ability of different TRAP variants with these metal ions is summarized in **Table** **1**. These results indicated that appropriate selection of the ligand number and structure are key for metal‐assisted TRAP‐cage assembly.

**Table 1:** The ability of various TRAP variants to form TRAP‐cages in the presence of different metal ions.

TRAP variant	Au(I)	Co(II)	Zn(II)
TRAP^K35C^	Y	N	Y
TRAP^S33H/K35C^	Y	N	Y
TRAP^S33H/K35H^	N	Y	Y*

Y = cages formed, N = cages not formed.*Cages were formed with low efficiency

### 2.3 Structures of TRAP‐Cages Formed with Co(II) and Zn(II)

**Figure 2:**
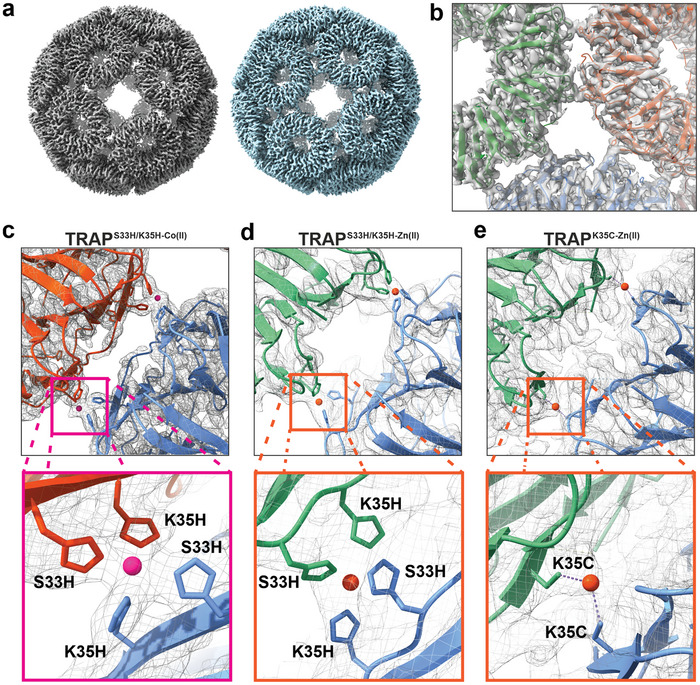
Structure of TRAP^S33H/K35H‐Co(II)^‐cage. a) CryoEM electron density maps of two reconstructed chiral forms of the TRAP^S33H/K35H‐Co(II)^‐cage. b) close‐up view of the interaction between rings in the cage. c) Enlarged view of the interface between two adjacent TRAP rings from TRAP^S33H/K35H‐Co(II)^‐cage with visible coordinated two cobalt (II) ions (pink spheres). d) detailed view of a ring‐ring interface from TRAP^S33H/K35H‐Zn(II)^‐cage centered on one of the zinc (II) ions (orange). e) detailed view of a ring‐ring interface from TRAP^K35C‐Zn(II)^‐cage centered on zinc (II) ion (orange). Residues involved in the coordination of metal ions are shown in stick representation. Protein is represented in ribbon format, reconstructed density is represented as a surface (panels a and b) or as a mesh (panels c‐e) contoured at RMSD = 3.5.

We constructured a number of cages named as follows: Cages made with Zn(II) (TRAP^K35C‐Zn(II)^‐cages); TRAP^S33H/K35H^ with either Co(II) or Zn(II) (TRAP^S33H/K35H‐Co(II)^ ^or^ ^‐Zn(II)^‐cages); TRAP^S33H/K35C^ with Zn(II) (TRAP^S33H/K35C‐Zn(II)^‐cages). To verify our designs, we solved the structures of TRAP^S33H/K35H‐Zn(II)^‐cage, TRAP^S33H/K35H‐Co(II)^‐cage, and TRAP^K35C‐Zn(II)^‐cages using cryoEM single particle reconstruction (see Methods for sample preparation and Figures S2–S4 (Supporting Information) for analysis pipelines). The resulting structures (**Figure** **2**) showed that these cages are mainly composed of 24 TRAP rings, and the overall shape and bonding network between adjacent rings is retained when compared to the TRAP^Au(I)^_24_‐cage.^[^ ^12^ ^]^ They form two chiral cages (Figure 2a), and each ring forms two metal‐mediated bridges with each neighbor (Figure 2b). Electrothermal atomic absorption spectroscopy (ETAAS) analysis of TRAP^S33H/K35H‐Co(II)^ and TRAP^K35C‐Zn(II)^‐cages showed an average of 118 and 114 metal ions per cage, respectively (**Table** **2**), close to the number of metal ions in the cryoEM‐solved structures with the maximum saturation, 120.

**Table 2:** Metal content of TRAP‐cages. Results of ETAAS analysis showing the number of indicted metal ions per cage.

TRAP variant	Metal	Metal content (mg/g)	SD	No. of ions per TRAP‐cage	SD
S33H/K35H	Co	3.55	0.12	118	4
S33H/K35H	Co	3.42	0.14	114	5
S33H/K35H	Co	3.63	0.17	121	6
K35C	Zn	3.23	0.04	119	1
K35C	Zn	2.93	0.04	108	1

In the cryoEM analysis, for TRAP^S33H/K35H‐Zn(II)^‐cages, considering both levo and dextro forms combined (Figure S5, Supporting Information), only ≈5% of the particles used in the ab initio reconstruction were suitable for use in the final 3D refinement. In contrast, ≈60% or 72% of particles were used in 3D refinement of TRAP^S33H/K35H‐Co(II)^‐ and TRAP^K35C‐Zn(II)^ – cages respectively. These results suggest that TRAP^S33H/K35H‐Zn(II)^ ‐cages had poor homogeneity compared to TRAP^S33H/K35H‐Co(II)^‐cages which were themselves somewhat less homogenous than TRAP^K35C‐Zn(II)^‐cages.

In the structure of TRAP^S33H/K35H‐Co(II)^‐cage (Figure 2c; Figure S6, Supporting Information), every bridge has in its center one ion in close proximity to the histidine residues introduced in positions 33 and 35 as expected (Figure 2c). However, a closer analysis revealed that the coordination geometry is unlikely to be a perfect tetrahedron but rather a distorted one or an octahedron formed by 4 histidine ligands from the protein and 2 bonds with other ligands such as water molecules (which are not visible due to limited resolution). While the exact coordination geometry was undetermined due to the limited resolution, cryoEM density maps of TRAP^S33H/K35H‐Zn(II)^ and TRAP^K35C‐Zn(II)^‐cages (Figures 2d,e and S7 and S8, Supporting Information) suggests the side chains of these introduced amino acids bind Zn(II) as expected (Figure 2d,e). The observed distances between metal and coordinating residues are within expected ranges and resembling that seen for Au(I) induced TRAP cages.

### 2.4 Stability of Zn(II) and Co(II)‐Mediated TRAP‐Cages

**Figure 3:**
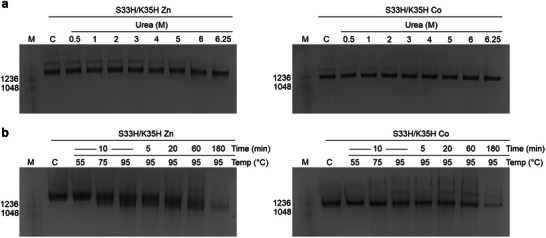
Stability of TRAP^S33H/K35H‐Co(II)^ and TRAP^S33H/K35H‐Zn(II)^ cages to urea and temperature. Native PAGE results are shown for stability a) After incubation in presence of increasing amounts of urea as indicated and b) After exposure to indicated temperatures for indicated times. Text on left of gels shows the position of molecular weight markers with molecular weights in kDa. “C” = control lane, i.e., untreated TRAP‐cages. For full range of stability tests see Figures S9 and 10 (Supporting Information).

**Figure 4:**
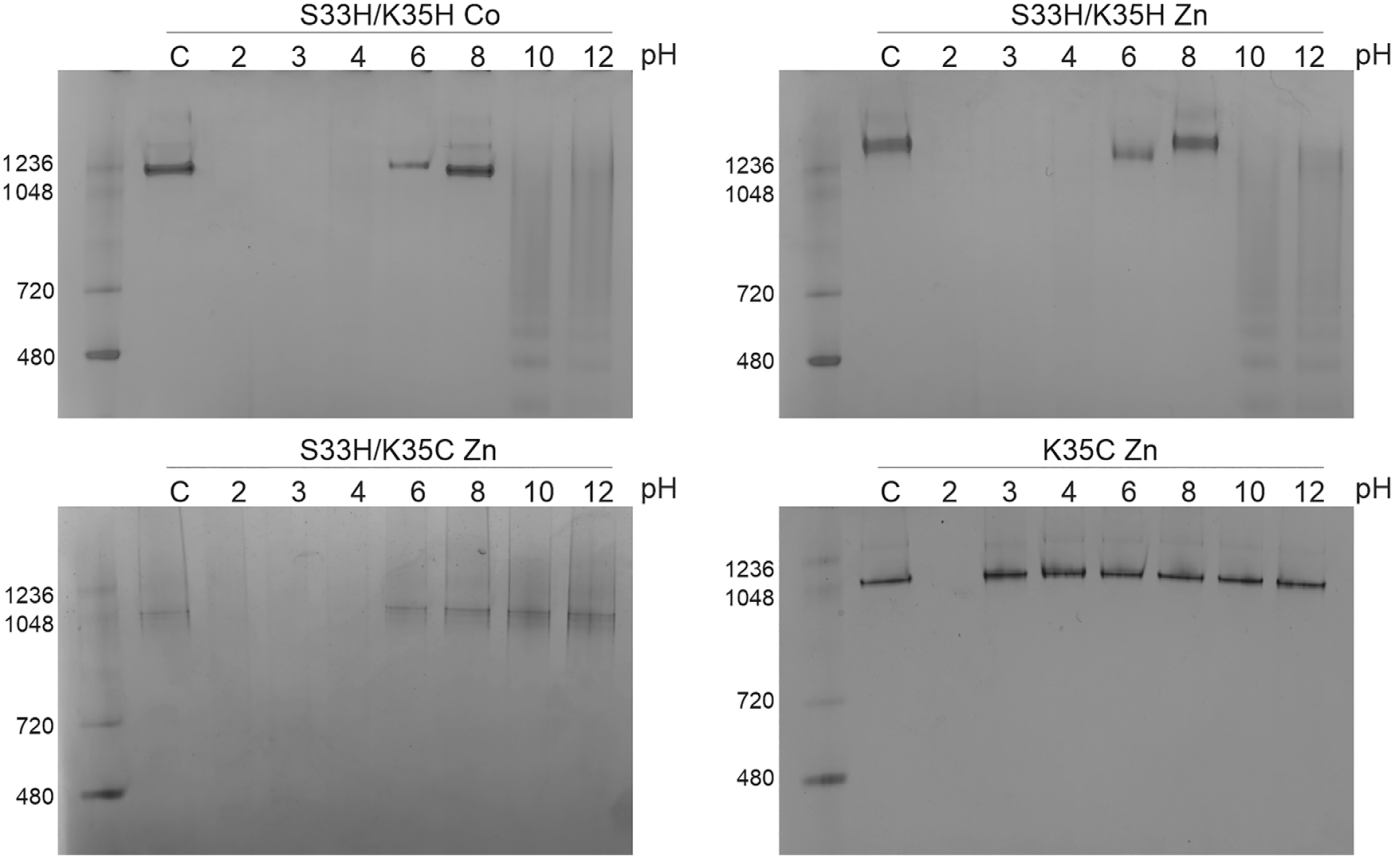
Effect of pH on cage stability. Native PAGE of TRAP^S33H/K35H‐Co(II)^, TRAP^S33H/K35H‐Zn(II)^, TRAP^S33H/K35C‐Zn(II)^, TRAP^K35C‐Zn(II)^ showing stability at different pH (as indicated).

TRAP^Au(I)^‐cages showed high stability with resistance to disassembly at temperatures >95 °C, pH extremes, and high concentrations of surfactants and chaotropic agents.^[^ ^12^ ^]^ To investigate if those TRAP‐cages formed by different variants and metal ions had significantly altered stability, we carried out a series of stability tests with TRAP‐cages constructed in the presence of different metals (**Figures** **3** and **4**, results summarised in **Table** **3**; Figures S9 and S10, Supporting Information).

**Table 3:** Summary of stability test results for TRAP‐cages constructed from TRAP variants as indicated. All results are from the present study unless otherwise indicated.

Assay	S33H/K35H‐Co(II)	S33H/K35H‐Zn(II)	S33H/K35C‐Zn(II)	K35C‐Zn (II)	K35C‐Au(I)
Temp (°C, 60 min)	≥95	≥95	≥95	≥95	≥95 ^[^ ^12^ ^]^
Urea (M)	≥6.25	≥6.25	≥6.25	≥6.25	≥7 ^[^ ^12^ ^]^
GdHCl (M)	>1	>0.5	>1	>1	>2^[^ ^12^ ^]^
SDS (%)	0.05–0.5	0.05–0.5	0.05–0.5	>5	>5^[^ ^12^ ^]^
GSH (mM)	3.5–35	≈3.5	3.5–35	3.5–35	>0.7 mM^[^ ^12^ ^]^
GSSG	35	>3.5–35	≥35	≥35	≥70 ^[^ ^12^ ^]^
DTT (mM)	0.35–3.5	0.35–3.5	0.35–3.5	0.35–3.5	0.7–7^[^ ^12^ ^]^
TCEP (mM)	35	≥35	≥35	≥35	0.07–0.7^[^ ^12^ ^]^
EDTA (mM)	0.0035–0.035	0.0035–0.035	0.0035–0.035	≥35*	≥35
pH	<4–6, >8–10	<4–6, >8–10	<4‐6, >12	<3,>12	<3, >12^[^ ^12^ ^]^

Entries underlined and in bold indicate the limit of the conditions tested and that the cage showed no disassembly. * Slow disassembly is visible only after 1–4 days.

Like TRAP^Au(I)^‐cages, all the tested TRAP‐cage assemblies exhibited resistance to high temperatures, at 95 °C for 180 min, and high urea concentrations, at least up to 6.25 M. These results suggested little effect of these variants on protein folding stability. However, substantial cage disassembly was observed for all the TRAP‐cages with guanidium hydrochloride (GdHCl) and sodium dodecyl sulfate (SDS). Guanidium and sulfate groups of those compounds likely strip off the constituent metal ions from the TRAP‐cage walls, instead of denaturing protein folding. Indeed, thiol‐containing reducing agents, dithiothreitol (DTT) and a reduced form of glutathione (GSH) induced disassembly of all the tested TRAP‐cages. Notably, 100‐ to 1000‐fold excess of these competing compounds; 35 µM (in terms of monomer) TRAP versus 3.5–35 mm DTT or GSH, were required for the ligand substitution reaction. The bonding network accompanying cage formation likely stabilizes the metal‐cysteine/histidine coordination owing to the chelating effect.

The TRAP‐cage variants showed different patterns of resistance against competing compounds. This suggests a dependence on the relative bond strength of metal‐cysteine or histidine coordination compared to those with competing compounds. While TRAP^S33H/K35H^‐cages with both Zn(II) and Co(II) disassembled at ≈1 m GdHCl, those containing cysteine ligands and Zn(II), TRAP^S33H/K35C‐Zn(II)^‐ and TRAP^K35C‐Zn(II)^‐cages, showed (partially) assembled cages in the presence of 4 m GdHCl as evidenced by a corresponding band on native PAGE. The Zn(II)‐thiol bridges in the TRAP‐cage structure likely have higher resistance against competing guanidine ligands compared to Zn(II)‐ or Co(II)‐imidazole bonds. The opposite tendency in assembly stability was observed in response to addition of DTT: While TRAP^S33H/K35H‐Co(II)^‐cages largely retained their cage structure in presence of 3.5 mm DTT, the same conditions induced moderate disassembly of those containing Zn(II), the TRAP^S33H/K35C‐Zn(II)^‐, TRAP^S33H/K35H‐Zn(II)^‐, or complete disassembly for TRAP^K35C‐Zn(II)^‐cages.

The most critical difference in the response of cages to competing agents was observed with EDTA. TRAP‐cages that possess only histidine ligands, TRAP^S33H/K35H‐Co(II)^ cages, and TRAP^S33H/K35H‐Zn(II)^ cages, almost completely disassembled in the presence of 35 µM EDTA (1 equivalent to TRAP monomer). TRAP‐cages with both histidine and cysteine ligands behaved differently, TRAP^S33H/K35C‐Zn(II)^ showed more resistance and full disassembly was visible at 350 µm EDTA while TRAP^S33H/K35C‐Au(I)^ was not sensitive to EDTA at all (cage did not disassemble at 35 mm EDTA). Interestingly, TRAP^K35C‐Zn(II)^ cage immediately after purification was sensitive to EDTA at the level of TRAP^S33H/K35C‐Zn(II)^ cage but we noted that after a prolonged period it became resistant to the disassembly‐promoting action of the chelating agent.

Meanwhile, all the cages exhibited high resistance against the tested competitors containing multiple carboxylate groups, tris(2‐carboxyethyl)phosphine (TCEP), and oxidized glutathione (GSSG). The tertiary amines of EDTA may contribute to the disassembly of TRAP^S33H/K35H‐Co(II) and Zn(II)^ cages. Considering the stability constants of Co(II)‐ or Zn(II)‐EDTA complexes are both ≈16,^[^ ^29^ ^]^ these results confirmed that TRAP variants containing K35C mutation have an extremely high affinity for Zn(II) ions.

Stability tests of the TRAP‐cages at various pHs showed the same tendency as observed in the EDTA titration experiments (Figure 4; Figure S10a,d, Supporting Information). Consistent morphology under a wide range of pHs (3–12) was observed for the TRAP^K35C‐Zn(II)^‐cage, similar to that seen for the TRAP^K35C‐Au(I)^‐cage. However, TRAP^S33H/K35C‐Zn(II)^‐cages appeared to begin to disassemble at pHs in the 6–8 range and lower. TRAP^S33H/K35H‐Co(II) and Zn(II)^‐cages were more sensitive to pH, disassembling completely at pHs below 4–6 and above 8–10. These extreme pHs likely compete with metal‐TRAP coordination, where low pH protonates those ligands and high pH hydrolyzes metal ions to form poorly soluble hydroxide products, the ability of TRAP^S33H/K35C‐Zn(II)^‐cages to withstand higher pHs compared to the double histidine variant likely reflecting the increased stability provided by coordination via the thiolates of the introduced cysteines. Together with the competitor titration experiments, these results suggested that the metal‐protein complex stability is in the order of TRAP^K35C‐Zn(II) –^ > TRAP^S33H/K35C‐Zn(II)^‐ > TRAP^S33H/K35H‐Zn(II)^‐ ≈ TRAP^S33H/K35H‐Co(II)^‐cages.

### 2.5 Reversible Triggering of Cage Opening

**Figure 5:**
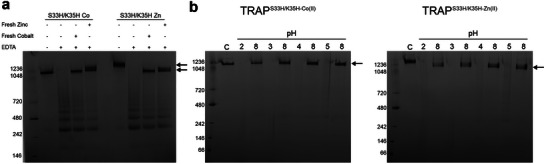
Reversibly triggered cage opening. a) Native PAGE of TRAP^S33H/K35H‐Co(II)^ and TRAP^S33H/K35H‐Zn(II)^ disassembly triggered by EDTA and reforming of the cage upon addition of excess cobalt or zinc ions. Arrows show position of TRAP‐cages. b) Native PAGE of TRAP^S33H/K35H‐Co(II)^ and TRAP^S33H/K35H‐Zn(II)^ disassembly triggered by low pH and back cage formation by increasing pH. Lanes labeled 2,3,4, and 5 show TRAP‐cage sample after incubation at the indicated pHs. Lanes labeled 8 are those samples after pH was increased to pH 8.

We found that EDTA‐mediated TRAP‐cage disassembly is reversible. Both TRAP ^S33H/K35H‐Co(II)^ and TRAP^S33H/K35H‐Zn(II)^ cages are held together by bridging cobalt and zinc ions respectively. The addition of EDTA removes the cobalt and zinc leading to disassembly of the cages. Subsequent addition of excess cobalt or zinc allows the cages to reform (**Figure** **5**a).

Triggering cage opening via change in pH was also tested. We hypothesized that lowering pH to <6.5 should protonate the imidazoles of coordinating histidines causing loss of coordination ability and subsequent cage disassembly. Results showed that this was indeed the case with fully formed cages as indicated by native PAGE, with cages being present at pH 8 but being largely disassembled at pH 6 and no cages being visible at pH 5 and below (Figure 5b).

## 3 Discussion

A useful feature of many protein cages is their high stability. This means that they have the ability to act as protective containers for cargoes that may otherwise be too fragile to survive particular conditions. Notably this includes cell culture or in vivo environments where potential macromolecular therapeutics such as functional folded proteins or nucleic acids that are not protected have a high probability of being rendered non‐functional before reaching their desired targets.

Protein cages are made from multiple proteins and typically held in place through protein‐protein interactions. These offer advantages of high stability but their diversity means that triggering disassembly (as required for cargo release) is challenging. Here, simpler types of connections between the proteins may be advantageous and include metal‐mediated bonds.

Control of protein‐cage disassembly has previously been shown for cages linked with Cu(II)^[^ ^8^ ^]^ where it is achieved by the use of a chelating agent (EDTA); Fe(II) and Zn(II)_;_^[^ ^9^ ^]^ Au(I)^[^ ^12^ ^]^ (disassembled by reducing agents); and chemical cross–linkers such as DTME^[^ ^24^ ^]^ (disassembled in reducing conditions). In this work we expanded the library of triggerable cross‐linker agents for TRAP‐cage, demonstrating Ag(I), Cd(II), Co(II), and Zn(II)‐linked cages. In the latter two, we re‐engineered the protein‐protein interfaces to change the metal binding site geometry. We removed the single cysteines in each TRAP monomer that formed linear coordinate bonds with gold. Replacement in a similar position on the TRAP ring with two His residues per monomer appears to provide the basis for tetrahedral/octahedral coordination of metals with water molecules likely providing additional coordination. Notably the redesign process allowed us to produce variants of TRAP that are able to form cages with cobalt, something which does not occur with previous iterations of the protein and may be advantageous in terms of safety and cost compared to previously demonstrated metals.

It is interesting to note the single cysteine variant of TRAP (K35C) was also able to form cages in the presence of Zn. It is possible that in this case there may be involvement of both the cysteine and other nearby residues. Though precise details regarding interactions will require further high‐resolution structural studies.

Changing the identity of the metal “glue” in this way has a significant effect on the programmable dynamics of a given protein cage as long as the cage has few or no other interactions holding the fundamental subunit building blocks in place, as is the case for TRAP‐cages. Consequently, the metal bridges alone determine the disassembly characteristics of the cage providing a simple and modular system for programming disassembly properties.

The lack of involvement for protein‐protein interactions also gives great freedom to redesign the protein to interact with different metals while still retaining overall structure and cage forming capability, aspects demonstrated by the new cages presented in this work.

Incorporation of His into the metal coordination site also allowed us to tune pH responsiveness of cage disassembly. Modified cages were sensitive to pHs below ≈7 which might be useful for therapeutic applications. For example, acidic pH is commonly found in tumor‐associated environments^[^ ^30^ ^]^ raising the prospect of TRAP‐cages that release cargoes only in these specific areas. Additionally, acidic pH is present inside cell endosome and lysosome which are also targets for intracellular drug delivery.^[^ ^31^ ^]^

Overall, our results, when combined with previous work showing guest packing,^[^ ^26^ ^]^ cell delivery,^[^ ^26^ ^]^ and external decoration^[^ ^26^ ^]^ are a further step toward development of TRAP‐cages as a useful platform for targeted smart delivery/display systems.

## Conflict of Interest

The authors declare the following competing financial interests: Y.A., K.M., A.P.B., S.G., and J.G.H. are named as inventors on a number of patent applications related to TRAP‐cage assembly, decoration, and filling. J.G.H. is also the founder of and holds equity in nCage Therapeutics, which aims to commercialize protein cages for therapeutic applications. S.G. is an employee of nCage Therapeutics.

## Author Contributions

J.G.H., Y.A., and A.P.B. conceptualized the research hypothesis. N.O., K.M., and S.G. performed protein production, purification, and biochemical characterization. Z.P.S. performed biochemical characterization. A.P.B. performed cryo‐EM experiments and structural analysis. JGH obtained funds. All authors contributed to the manuscript writing.

## Supporting information



Supporting Information

## Data Availability

The data that support the findings of this study are available from the corresponding author upon reasonable request. Original gel images have been deposited at Mendeley and are publicly available as of the date of publication (DOI: 10.17632/nprxj3dmvm.1).
