# Supplementary material for: Designed, Programmable Protein Cages Utilizing Diverse Metal Coordination Geometries Show Reversible, pH‐Dependent Assembly
Source: Macromol Rapid Commun. 2024 Dec 16;46(6):2400712. doi: 10.1002/marc.202400712 (PMC11925324; doi:10.1002/marc.202400712)
Supplement: Supplementary file 1 — Supporting Information [file MARC-46-2400712-s001.pdf]

Macromolecular Rapid Communications

Supporting Information

for *Macromol. Rapid Commun.*, DOI 10.1002/marc.202400712

Designed, Programmable Protein Cages Utilizing Diverse Metal Coordination Geometries Show Reversible, pH-Dependent Assembly

*Norbert Osiński, Karolina Majsterkiewicz, Zuzanna Pakosz-Stępień, Yusuke Azuma, Artur P. Biela, Szymon Gaweł and Jonathan G. Heddle**

SUPPORTING INFORMATION

Designed, Programmable Protein Cages Utilizing Diverse Metal Coordination Geometries Show Reversible, pH-Dependent Assembly

Norbert Osiński[1,3], Karolina Majsterkiewicz[1,2], Zuzanna Pakosz-Stępień[1], Yusuke Azuma[1], Artur P. Biela[1,2], Szymon Gaweł[1,3], & Jonathan G. Heddle[1,4]

[1]Malopolska Centre of Biotechnology, Jagiellonian University, Gronostajowa 7A, Kraków, 30-387, Poland

[2]Postgraduate School of Molecular Medicine, ul. Żwirki i Wigury 61, Warsaw 02-091, Poland

[3]Doctoral School of Exact and Natural Sciences, Jagiellonian University, Łojasiewicza 11, Kraków, 30-384, Poland

---

[1] School of Biological and Biomedical Sciences, Durham University, Durham DH1 3LE, UK

[2] National Synchrotron Radiation Centre SOLARIS, Czerwone Maki 98, Kraków, 30-392, Poland

Correspondence to: Jonathan.g.heddle@durham.ac.uk

Contents

1. Supporting Data 3

Figure S1 3

Figure S2 4

Figure S3 5

Figure S4 6

Figure S5 7

Figure S6 8

Figure S7 9

Figure S8 10

Figure S9 11

Figure S10 12

2. Materials and Methods 13

Plasmids and molecular cloning 13

Table S1. Plasmids and amino acid sequences 14

Table S2. Sequences of primers 15

Protein Expression and Purification 15

Native-PAGE 16

Cage Assembly With Different Metals 16

Stability Assays 17

EDTA and pH Dependent Reversible Cage Disassembly 17

Transmission Electron Microscopy 17

CryoEM Reconstruction 18

Quantitation of Metal Content 19

Table S3: CryoEM Statistics 20

1. Supporting Data

Figure S1

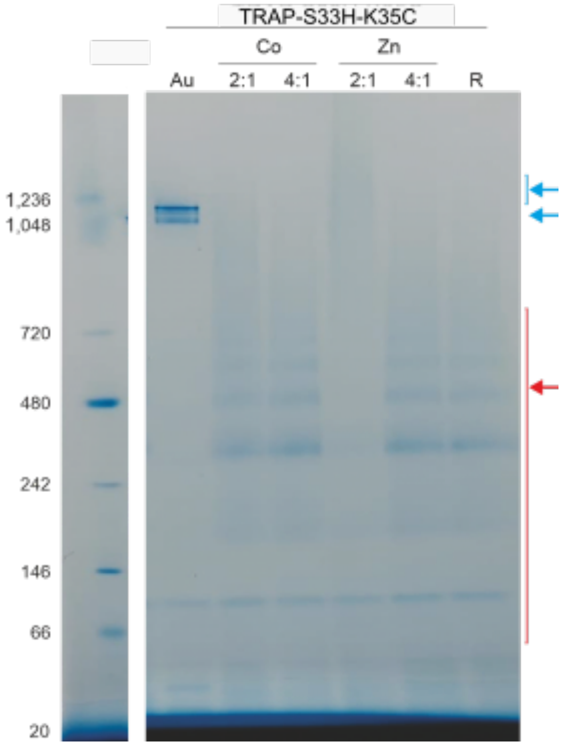

Supplementary Figure 1. Native-PAGE gel showing behavior of the TRAP$^{S33H/K35C}$ in the presence of different metal ions. Addition of Au(I), Co(II) and Zn(II) clearly shows the capability of the protein to form cages with gold and zinc ions. TRAP$^{S33H/K35C}$ monomer and cage band are marked with arrowheads in red and blue respectively. Marked ratios indicate relative concentrations of TRAP monomer to indicated metals.

Figure S2

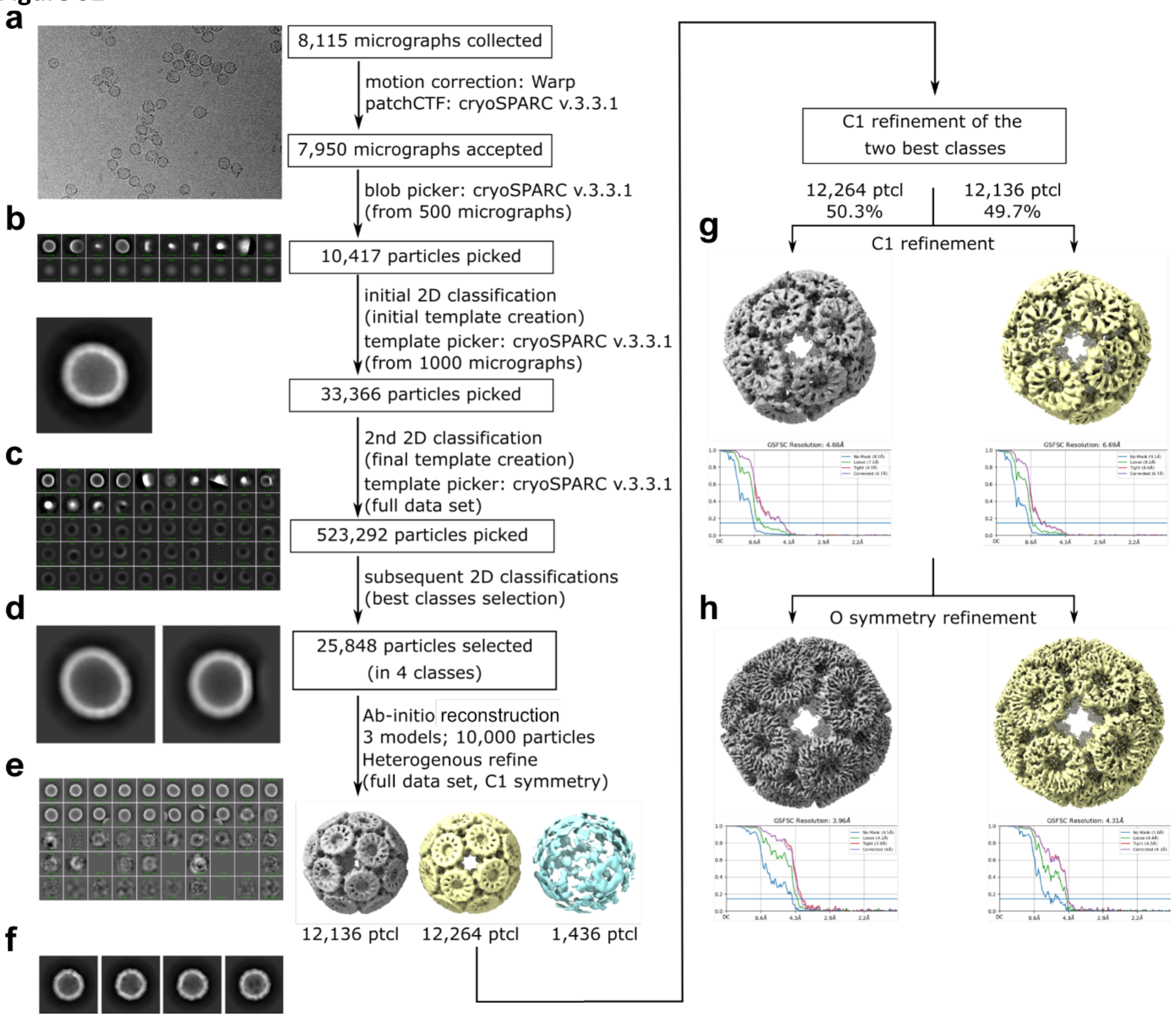

Supplementary Figure 2. TRAP$^{S33H/K35H-Zn(II)}$–cage cryoEM reconstruction pipeline: a,example micrograph (scale bar 50 nm), **b,** first reference-free 2D classes, **c,** selected class used as a first template, **d,** reference-free 2D classes after first template pick on 1000 micrographs, **e,** selected 2D classes for final template picking, **f,** final reference-free 2D classification, used for 3D reconstruction, **g,** C1-symmetry refined two chiral forms of TRAP$^{S33H/K35H-Zn(II)}$-cages, **h,** final O-symmetry refined two chiral forms of TRAP$_{S33H/K35H-Zn(II)}$-cages

Figure S3

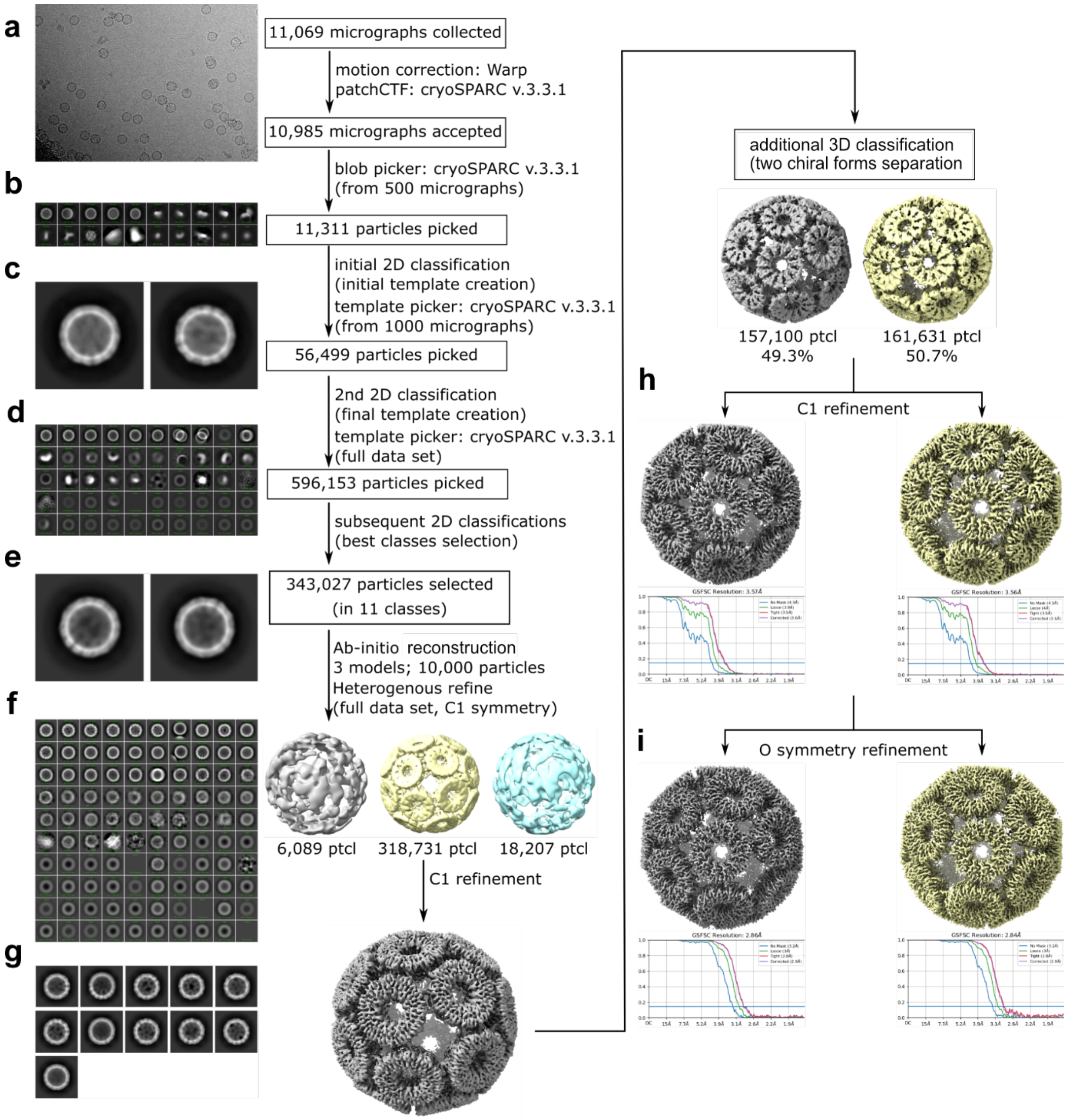

Supplementary Figure 3. TRAP$^{S33H/K35H\text{-}Co}$-cage cryoEM reconstruction pipeline: **a,** example micrograph (scale bar 50 nm), **b,** first reference-free 2D classes, **c** selected classes used as a first template, **d** reference-free 2D classes after first template pick on 1000 micrographs, **e,** selected 2D classes for final template picking, **f,** final reference-free 2D classification, **g,** final 2D classes selected for 3D reconstruction, **h,** C1-symmetry refined two chiral forms of TRAP$^{S33H/K35H\text{-}Co}$-cages, **i,** final Osymmetry refined two chiral forms of TRAP$^{S33H/K35H\text{-}Co}$-cages

Figure S4

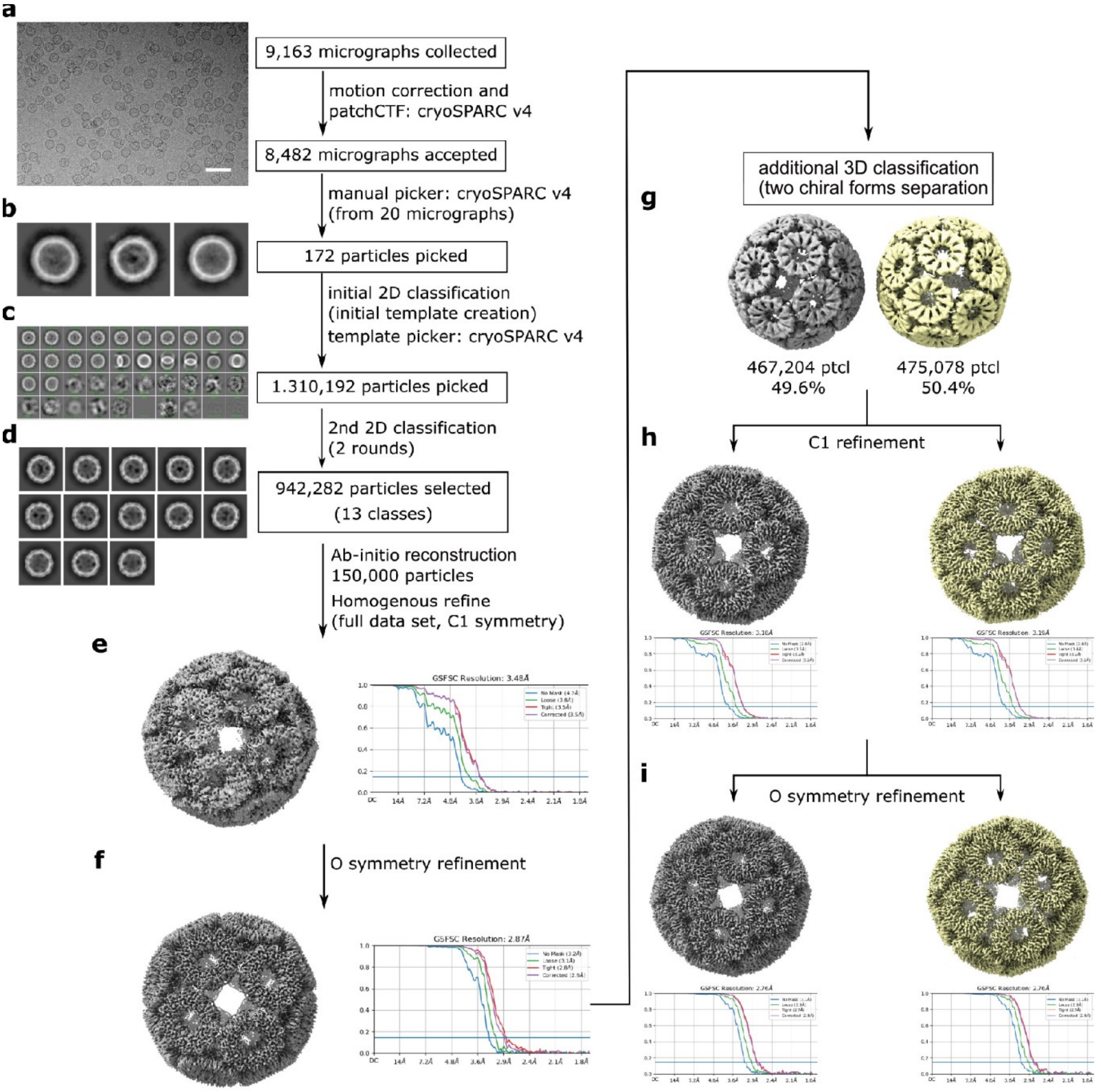

Supplementary Figure 4. TRAP$^{K35C\text{-}Zn(II)}$-cage cryoEM reconstruction pipeline: a, example micrograph (scale bar 50 nm), **b.** first reference-free 2D classes, **c** selected class used as a first template, **d,** reference-free 2D classes after first template pick on 1000 micrographs, **e,** selected 2D classes for final template picking, **f,** final reference-free 2D classification, **g,** final 2D classes selected for 3D reconstruction, **h,** C1-symmetry refined two chiral forms of TRAP$^{K35C\text{-}Zn(II)}$-cages, **i** final O-symmetry refined two chiral forms of TRAP$^{K35C\text{-}Zn(II)}$-cages.

Figure S5

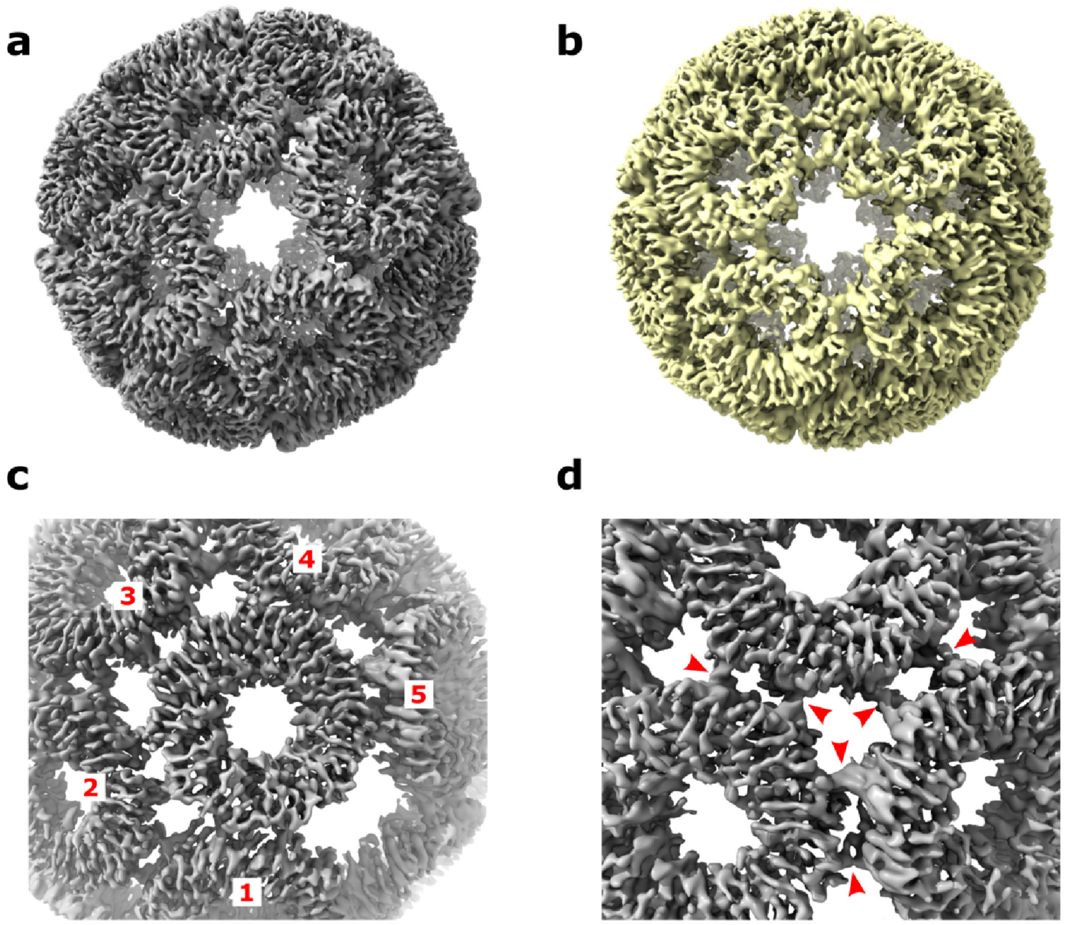

Supplementary Figure 5. Two chiral forms TRAP$^{S33H/K35H\text{-}Zn(II)}$ cages. a and **b** show chiralA and chiralB respectively. **c**, close-up view centered on one ring showing 5 neighbors (marked with red numbers), **d**, close-up view on the connections between rings, showing two bridges (marked with red arrowheads) formed between adjacent rings.

Figure S6

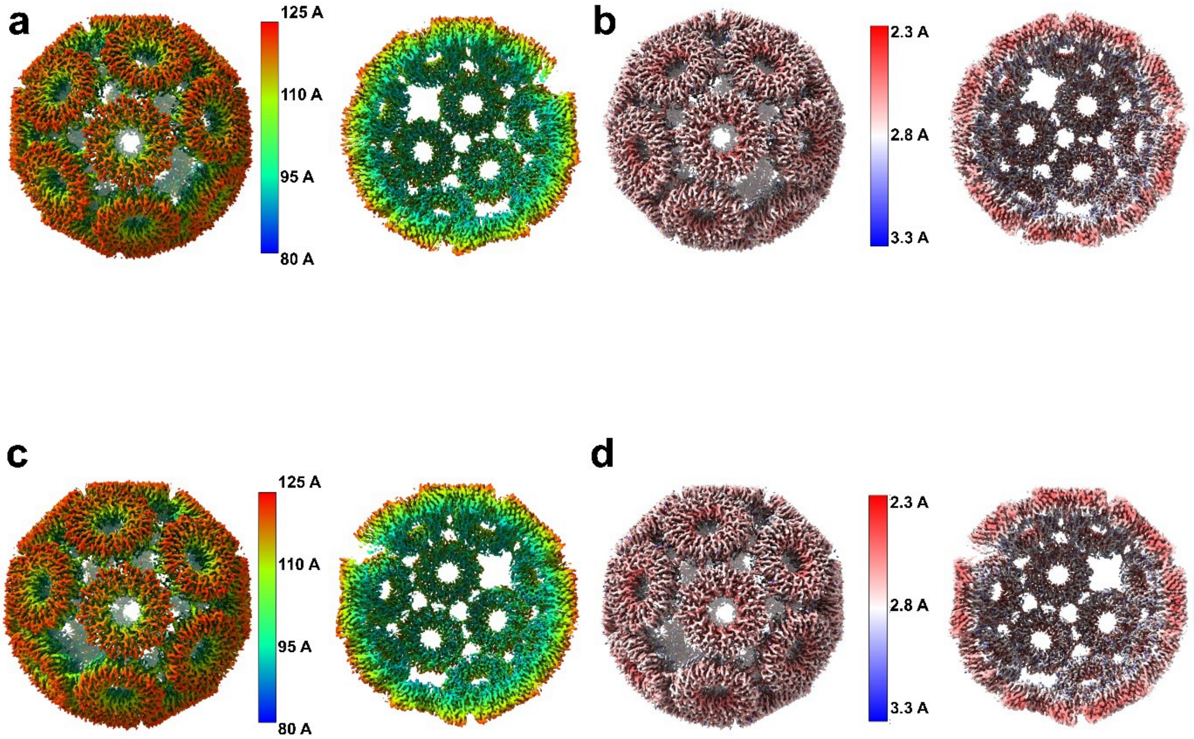

Supplementary Figure 6. TRAP$^{S33H/K35H-Co}$-cages in two representations: a, b – chiralA, colored by radius and local resolution respectively, **c, d** – chiralB, colored by radius and local resolution respectively; the left panel represent the surface view and right panels represent a cross-section view of respective cage.

Figure S7

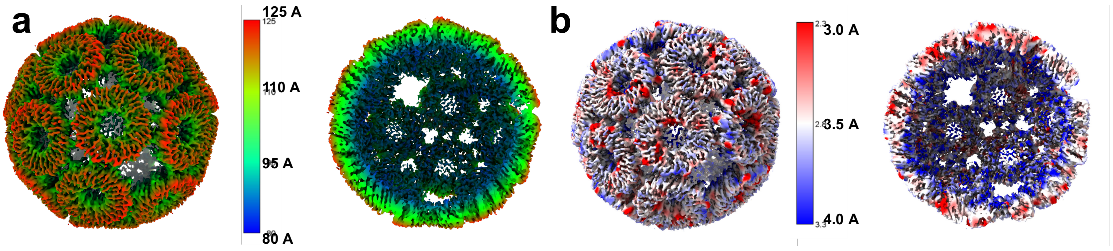

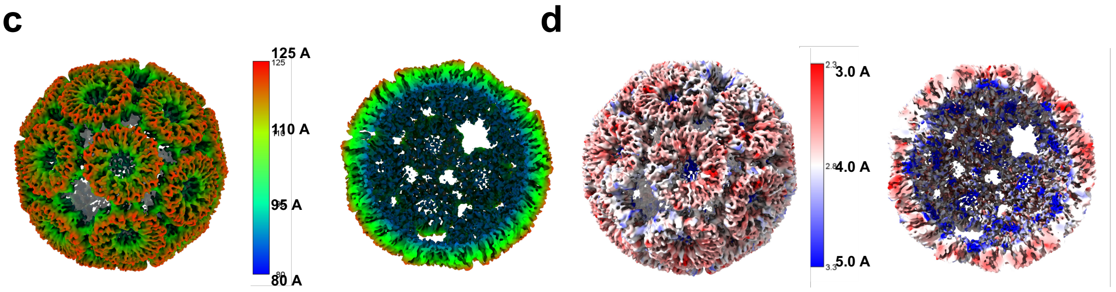

Supplementary Figure 7. TRAP$^{S33H/K35H\text{-}Zn(II)}$-cages in two representations: a,b – chiralA, colored by radius and local resolution respectively, **c, d** – chiralB, colored by radius and local resolution respectively; the left panel represent the surface view and right panels represent a cross-section view of respective cage.

Figure S8

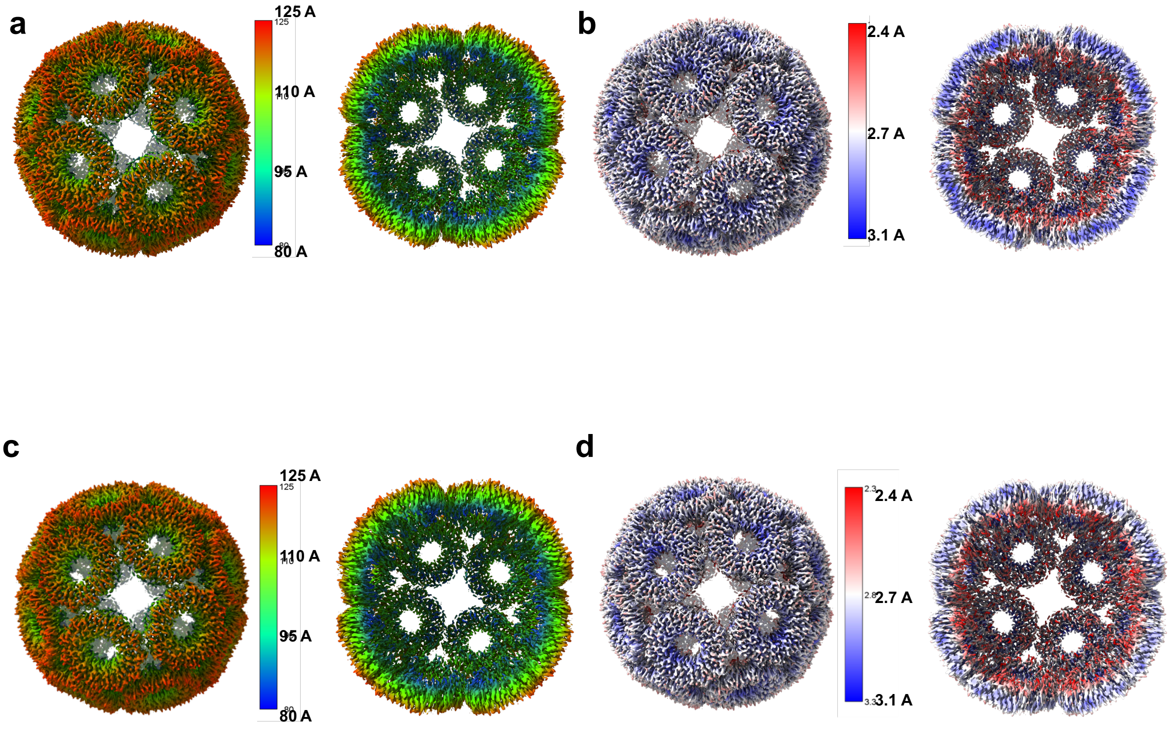

Supplementary Figure 8. TRAP$^{K35C\text{-}Zn(II)}$-cages in two representations: a,b – chiralA, colored by radius and local resolution respectively, **c, d** – chiralB, colored by radius and local resolution respectively; the left panel represent the surface view and right panels represent a cross-section view of respective cage.

Figure S9

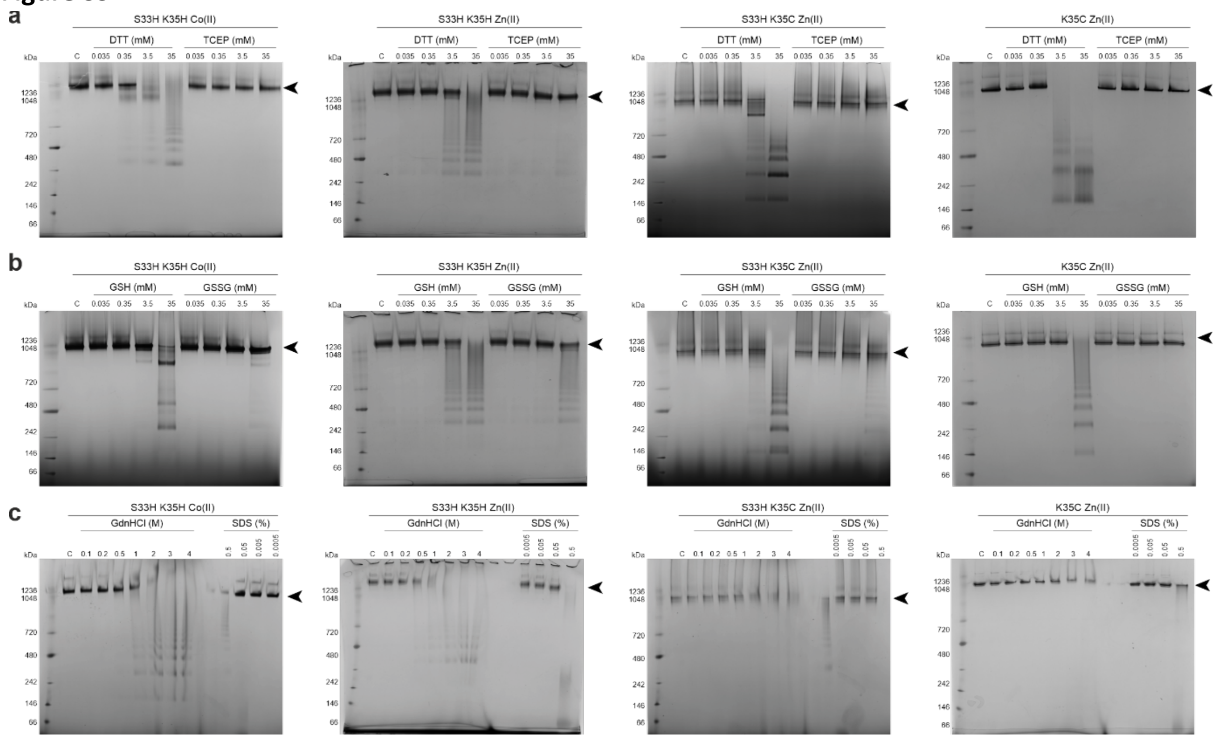

Supplementary Figure 9. Native-PAGE gels probing the stability of the TRAP-cages upon incubation with **a,** DTT and TCEP, **b,** GSH and GSSG, **c,** GndHCl and SDS. In each case protein cage variant identity is indicated above the corresponding gel together with the tested conditions/compound. Black arrowheads point to the intact cage band on the gel.

Figure S10

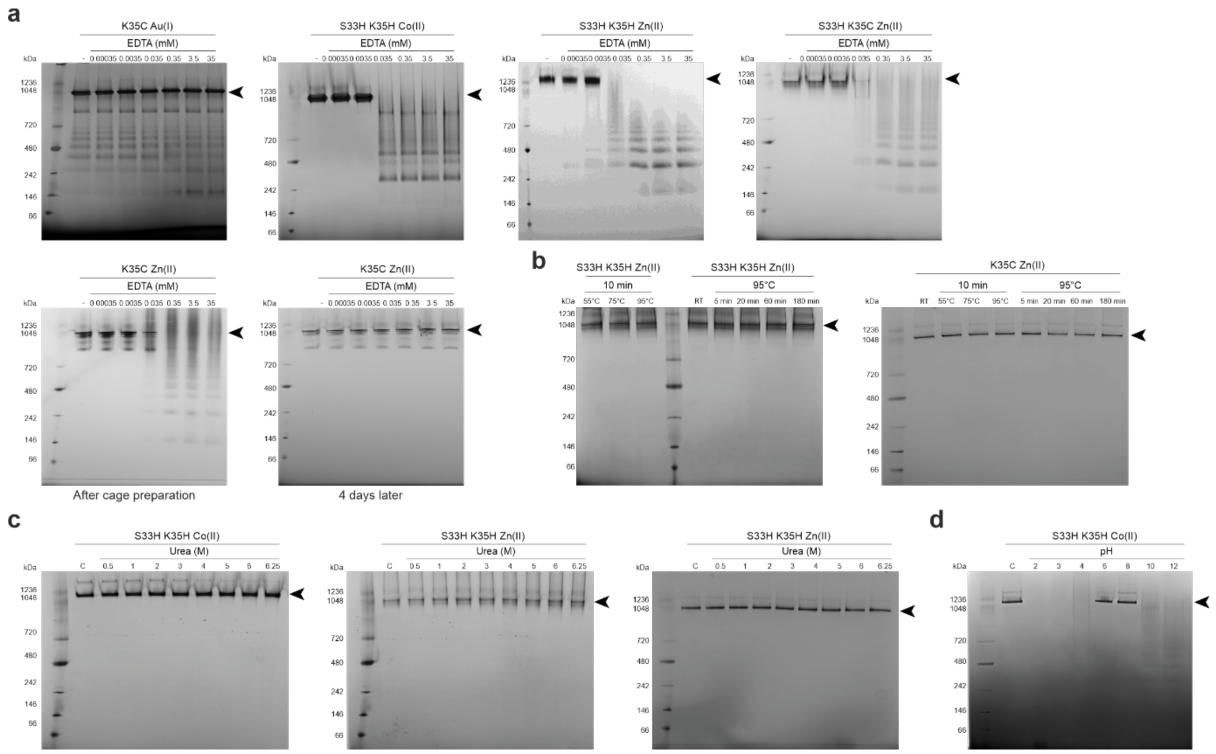

Supplementary Figure 10. Native-PAGE gels represent the stability of TRAP protein cages upon incubation with **a,** EDTA, **b,** at high temperature, **c,** Urea and **d,** pH. In each case protein cage variant identity is marked above the corresponding gel together with the tested conditions/compound. Black arrowheads point to the intact cage band on the gel.

2. Materials and Methods

Plasmids and molecular cloning

Details of produced proteins and relevant expression vectors are listed in **Table S1**. pET21b_TRAPK35C (synthesized by BioCat GmbH) was used to prepare expression vectors carrying genes encoding TRAP variants. Inserts encoding TRAP variants were subcloned in place of the TRAP(K35C) gene. The genes for TRAP(K35H), TRAP(S33H/K35H), TRAP(S33H/K35C) were amplified by PCR using pET21b_TRAP-K35H-H, pET21b_TRAP-S33H-K35H-H, pET21b_TRAP-S33H-K35C-H as templates respectively and oligonucleotides, FW_NdeI_TRAP and RV_XhoI_stop_TRAP2 (see **Table S2**) as primers. Primer RV_XhoI_stop_TRAP2 was constructed to introduce a stop codon.

The gene for TRAP(K35C) was amplified by PCR using pET21b_TRAP-K35C as template and

oligonucleotides, FW_NdeI_TRAP and RV_XhoI_TRAP (see **Table S2**) as primers.

The K35H mutation was introduced via a QuickChange (Agilent) reaction using pET21b_TRAP-K35C-H as template and FW_TRAP-K35H and RV_TRAP-K35H (see **Table S2**) as primers.

Similarly, pET21b_TRAP-S33H-K35H-H (pET21b_TRAP-S33H-K35C-H) was prepared in QuickChange reaction using pET21b_TRAP-K35H-H (pET21b_TRAP-K35C-H) as template and FW_TRAP-S33H-K35H (FW_TRAP-S33H-K35C) and RV_TRAP-S33H-K35H (RV_TRAP-S33H-K35C) (see **Table S2**) as primers.

E. coli NEB 5-alpha strain (New England Biolabs) was used for all the cloning steps. Plasmid sequences were confirmed by Sanger sequencing performed by Eurofins Genomics. Phusion High-Fidelity DNA Polymerase, T4 DNA ligase, GeneJET Plasmid Miniprep Kit, GeneJET Gel Extraction Kit were purchased from Thermo Fisher Scientific. Restriction enzymes used for molecular cloning were purchased from the New England Biolabs.

Table S1. Plasmids and amino acid sequences. Plasmids encoding TRAP variants with His-tag contain "-H" in name. His-tag is highlighted in blue in the amino acid sequence. Amino acids introduced as metal binding sites are shown in red.

**Plasmid name**	**Protein of interest**	**Amino acid sequence**
pET21b_TRAP-K35C	TRAP(K35C)	MYTNSDFVVIKALEDGVNVIGLTRGADTRFH HSE**C**LDKGEVLIAQFTEHTSAIKVRGKAYIQTR HGVIESEGKK
pET21b_TRAP-K35H	TRAP(K35H)	MYTNSDFVVIKALEDGVNVIGLTRGADTRFH HSE**H**LDKGEVLIAQFTEHTSAIKVRGKAYIQTR HGVIESEGKK
pET21b_TRAP-S33H-K35H	TRAP(S33H/K35H)	MYTNSDFVVIKALEDGVNVIGLTRGADTRFH H**H**E**H**LDKGEVLIAQFTEHTSAIKVRGKAYIQT RHGVIESEGKK
pET21b_TRAP-S33H-K35C	TRAP(S33H/K35C)	MYTNSDFVVIKALEDGVNVIGLTRGADTRFH H**H**E**C**LDKGEVLIAQFTEHTSAIKVRGKAYIQT RHGVIESEGKK
pET21b_TRAP-K35C-H	TRAP(K35C)-H	MYTNSDFVVIKALEDGVNVIGLTRGADTRFH HSE**C**LDKGEVLIAQFTEHTSAIKVRGKAYIQTR HGVIESEGKKLE**HHHHHH**

pET21b_TRAP-K35H-H	TRAP(K35H)-H	MYTNSDFVVIKALEDGVNVIGLTRGADTRFH HSEHLDKGEVLIAQFTEHTSAIKVRGKAYIQTR HGVIESEGKKLEHHHHHH
pET21b_TRAP-S33H-K35H-H	TRAP(S33H/K35H)-H	MYTNSDFVVIKALEDGVNVIGLTRGADTRFH HHEHLDKGEVLIAQFTEHTSAIKVRGKAYIQT RHGVIESEGKKLEHHHHHH
pET21b_TRAP-S33H-K35C-H	TRAP(S33H/K35C)-H	MYTNSDFVVIKALEDGVNVIGLTRGADTRFH HHECLDKGEVLIAQFTEHTSAIKVRGKAYIQT RHGVIESEGKKLEHHHHHH

Table S2. Sequences of primers. Underlined sequences are complimentary to template vectors.

**Primer Name**	**Sequence 5'-3'**
FW_NdeI_TRAP	TATACATATGTACACCAACTCTGACTTC
RV_XhoI_stop_TRAP2	CACGCTCGAGTTATTTTTTACCTTCAGATTCGATAACACC
RV_XhoI_TRAP	GAGCCTCGAGTTTTTTACCTTCAGATTCGATAACACC
FW_TRAP-K35H	CCCGTTTCCACCACTCTGAACACCTGGACAAAGGTGAAGTTC
RV_TRAP-K35H	GAACTTCACCTTTGTCCAGGTGTTCAGAGTGGTGGAAACGGG
FW_TRAP-S33H-K35H	GACACCCGTTTCCACCACCATGAACACCTGGACAAAGGTG
RV_TRAP-S33H-K35H	CACCTTTGTCCAGGTGTTCATGGTGGTGGAAACGGGTGTC
FW_TRAP-S33H-K35C	GACACCCGTTTCCACCACCATGAATGCCTGGACAAAGGTG
RV_TRAP-S33H-K35C	CACCTTTGTCCAGGCATTCATGGTGGTGGAAACGGGTGTC

Protein Expression and Purification

TRAP(K35C/R64S) protein was expressed and purified as described previously[12].TRAP(K35C), TRAP(K35H), TRAP(S33H/K35C), and TRAP(S33H/K35H) proteins were expressed and purified according to the same protocol as previously described for TRAP(K35C/R64S)[12] except that all buffers were at pH 8.5. In a typical purification, *E. coli* BL21(DE3)-star cells (Thermo Scientific) transformed with pET21b plasmid harboring the relevant gene were grown at 37 °C with shaking in 1 L of LB medium with 100 µg/ml ampicillin until OD_{600} = ~0.6, at which point gene expression was induced with 0.5 mM isopropyl ß-$_{D}$-1-thiogalactopyranoside (IPTG) followed by further shaking for 4 h. Cells were harvested by centrifugation and the pellet kept at -80 °C until use. Cells were lysed by sonication on ice in 50 ml of 50 mM Tris-HCl, pH 8.5, 50 mM NaCl, in the presence of proteinase inhibitors (Roche) and 2 mM

DTT, and lysates were centrifuged at 66,063 ×g for 0.5 h at 4 °C. The supernatant fraction was heated at 70 °C for 10 min, cooled to 4 °C, and centrifuged again at 66,063 g for 0.5 h at 4 °C. The supernatant fraction was loaded on 4 × 5 ml HiTrap QFF columns (GE Healthcare) attached to an ÄKTA Start (GE Healthcare) with binding in 50 mM Tris-HCl, pH 8.5, 0.05 M NaCl, 2 mM DTT buffer and eluting with a 0.05 - 1 M NaCl gradient. Fractions containing TRAP were pooled, concentrated using Amicon Ultra 30 kDa MWCO centrifugal filter units (Merck-Millipore), and subsequently subjected to size exclusion chromatography on a HiLoad 16/600 Superdex 200 pg column (GE Healthcare) in 50 mM Tris-HCl, pH 7.9, 0.15 M NaCl at room temperature. The eluted fractions were buffer exchanged using Amicon Ultra 30 kDa MWCO centrifugal filter units (Merck-Millipore) against the buffer: 50 mM Hepes-NaOH pH 7.9, 0.15 M NaCl. Protein concentration was determined by absorbance at 280 nm using a Nanodrop device (Thermo Fisher Scientific) with default settings assuming 1 absorbance unit corresponds to 1mg/ml of protein.

Native-PAGE

Samples were prepared in Native-PAGE loading buffer (50 mM Bis-Tris–HCl, pH 7.2, 0.1% [w/v] Bromophenol blue, 10% [w/v] glycerol). Next, samples were loaded onto gradient 3-12% Bis-Tris gels and resolved using NativePAGE Running buffer (Novex) at 150V for 90 min. To improve cage entering the gel Blue Native-PAGE was used, where cathode running buffer was additionally supplemented with 0.02% [w/v] Coomassie Brilliant Blue G-250. After the run gel was stained with Readyblue Protein Stain (Sigma-Aldrich). In case of Blue Native-PAGE, gel after run was incubated with fix solution (40% methanol, 10% acetic acid) for 15 minutes with shaking. Then with destain solution (8% acetic acid) to remove blue background. Gels were visualized with ChemiDoc MB (Bio-Rad).

Cage Assembly With Different Metals

Formation of TRAP-cages was carried out by mixing purified TRAP variants (final concentration of 0.1 mM with respect to monomeric subunits) with the relevant metal salt in a TRAP monomer : metal ion ratio of between 4:1 – 2:1 in suitable buffer: $AgNO_3$ in 50 mM Tris-HCl, pH 7.9, 0.15 M $NaNO_3$; $Cd(NO_3)_2$

in 50 mM Tris-HCl, pH 7.9, 0.15 M NaCl; $CoCl_2$ or $ZnCl_2$ in 50 mM HEPES-NaOH, pH 7.9, 0.15 M NaCl. Reactions were typically incubated for 3 days at room temperature. Formation of TRAP-cage was confirmed using native PAGE and TEM. Native PAGE sample buffer consisted of 50 mM Bis-Tris-HCl, pH 7.2, 0.125% [w/v] Bromophenol blue, 10% [w/v] glycerol. Any precipitated material was removed by centrifugation at 12,045 × g for 5 min.

Stability Assays

All the reagents used for cage stability assays (EDTA, DTT, TCEP, GSH, GSSG, SDS, GdnHCl, and urea) were reconstituted in water or cage buffer (50 mM HEPES pH 7.9, 150 mM NaCl) and the pH was adjusted when needed. Buffers used for pH stability assays were: 50 mM glycine-HCl at pH 2.0 or 3.0, 50 mM sodium acetate at pH 4.0, 50 mM potassium phosphate at pH 6.0, 50 mM HEPES-NaOH at pH 8.0, 50 mM glycine-NaOH at pH 10.0, 50 mM potassium phosphate at pH 12. Each sample was incubated overnight at room temperature. After brief centrifugation in a desktop centrifuge, the supernatant was mixed with 4 x native PAGE sample buffer and subjected to blue native PAGE. Cage stability experiments were repeated at least three times, each giving similar results.

EDTA and pH Dependent Reversible Cage Disassembly

Both $TRAP^{Co(II)}$ and $TRAP^{Zn(II)}$ cages (35 µM with respect to the TRAP monomer) were incubated overnight at room temperature with EDTA (35 µM) in the cage buffer to achieve disassembly. Next, additional cobalt or zinc was added to the final concentration of 17.5 µM, followed by incubation for 3 days for cage reassembly. For the pH-dependent cage disassembly, TRAP-cages (35 µM with respect to the TRAP monomer) were mixed with buffer (20 mM HEPES, 20 mM MES, and 20 mM sodium acetate) at different pHs (2, 3, 4, and 5). After overnight incubation at room temperature, 10 x cage buffer was added to the sample to give a final 1 x concentration and the sample was incubated for at least three days at room temperature.

Transmission Electron Microscopy

Samples were typically diluted to a final concentration of 0.05 mg/ml protein. After brief centrifugation, the supernatant was applied onto a glow-discharged carbon-coated copper grid (STEM). The samples were stained with 3% phosphotungstic acid, pH 8.0, and visualized on a JEOL JEM-1230 microscope operated at 80 kV.

CryoEM Reconstruction

All cryoEM-imaged samples were flash frozen in liquid ethane using a Vitrobot IV (Thermo Fisher Scientific). 4 mL of each protein cage sample were applied onto a copper grid (Quantifoil, Cu 2/1, mesh 300) and blotted for 4s (blot force 0, wait time 0s, drain time 0s) at 100% humidity at 4 °C. Frozen grids were imaged using TitanKrios (Thermo Fisher Scientific) microscope equipped with a K3 camera (Gatan) with ~40 e^{-}/A^{2} of total dose and magnification resulting in 0.86 Å/px. Typical data collection and refinement was as follows: Collected raw movies were first motion corrected using PatchMotionCorrection in cryoSPARC[26] and then the CTF function was estimated (PatchCTF/cryoSPARC). Particles were manually selected and subjected to reference-free 2D classification in order to produce the first set of templates. Template picking was done twice; firstly on 1000 micrographs, and after refining the templates, on a full set of data. After final 2D classification, selected particles were used to produce first 3D reconstructions (Ab-initio reconstruction/cryoSPARC). Best class was refined and 3D classified in order so separate two chiral forms (levo and dextro) of the produced cages. The separated forms of the protein cages were then refined (HomogenousRefinement/cryoSPARC) without any symmetry (C1) and next with enforcing octahedral symmetry (O) of the final assembly. All reconstructions were deposited in the Electron Microscopy Data Bank (EMDB) with codes as follows: S33HK35H-Co (EMD-18904, EMD-18905); S33HK35H-Zn (EMD-18906, EMD-18907); K35C-Zn (EMD-18908, EMD-18909) (**Table S3**).

Quantitation of Metal Content

Protein samples (0.35µM with respect to the TRAP monomer) were lyophilized and resolved in 0.2 or

1.0% HCl aqueous solution to the final protein concentrations (with respect to the TRAP monomer) of 2.8 μM or 12 μM for $TRAP^{S33H/K35H\text{-}Co(II)\ or\ Zn(II)}$-cages, respectively. Electrothermal atomic absorption (ETAA) spectra were measured on an AAS 3100 atomic absorption spectrometer (Perkin Elmer) at 240.73 nm (slit 0.2 nm, cobalt) or 213.86 nm (slit 0.7 nm, zinc), with deuterium background correction. These ions were quantified using a standard curve fitting to estimate metal-protein stoichiometry. Experiments were performed with 4 or 2 batches of TRAPS33H/K35H-Co(II) or $TRAP_{S33H/K35H\text{-}Zn(II)}$-cages, respectively, and means ± standard deviations calculated.

Table S3: CryoEM Statistics

Table summarises statistics for three different cage variants as indicated.

	S33HK35H-Co		*S33HK35H-Zn*		*K35C-Zn*	
EMDB/PDB id	EMD-18905/8R5A	EMD-18904/8R59	EMD-18907	EMD-18906	EMD-18909	EMD-18908
Data collection and processing						
Voltage (kV)	300	300	300	300	300	300
Electron exposure (e-/Å^2)	40	40	40	40	40	40
Defocus range (μm)	-1.0 ÷ -3.5	-1.0 ÷ -3.5	-1.0 ÷ -3.5	-1.0 ÷ -3.5	-1.0 ÷ -3.5	-1.0 ÷ -3.5
Symmetry imposed	O	O	O	O	O	O
Initial images (no.)	11069	11069	8155	8155	9163	9163
Final images (no.)	10985	10985	7950	7950	8482	8482
Final particles (no.)	161,631	157,100	12,264	12,136	467,204	475,078
Map resolution (Å)	2.84	2.86	3.96	4.31	2.76	2.76
FSC threshold	0.143	0.143	0.143	0.143	0.143	0.143
Map resolution range (Å)	1.86 ÷ 7.31	1.88 ÷ 7.31	2.91 ÷ 7.11	2.37 ÷ 6.28	2.44 ÷ 3.18	2.45 ÷ 3.21
Refinement						
Initial model used (PDB code)	4V4F	4V4F				
Model resolution	3.8	3.8				
Model composition						
Protein residues (no.)	18680	18680				
Ligands (no.)	120	120				
Validation						
RMS deviations						
Bond lengths (Å)	0.005	0.004				
Bond angles (°)	0.625	0.561				
MolProbity score	1.49	1.49				
Clash score	6.99	7.22				
Ramachandran plot						
	97.50	97.58				

Favored (%)	2.50	2.42
Allowed (%)		

Outliers (%)	0	0
Model/map fit (volume)		
CC (mask)	0.86	0.86
CC (box)	0.81	0.81
CC (peaks)	0.78	0.78
CC (volume)	0.83	0.82
Mean CC for ligands	0.89	0.89
